# Multi-Scale Fourier Temporal Network for Multi-Source Precipitation Nowcasting

**DOI:** 10.3390/s26082303

**Published:** 2026-04-08

**Authors:** Jing Huang, Shanmin Yang, Xiaojie Li, Xi Wu

**Affiliations:** School of Computer Science, Chengdu University of Information Technology, Chengdu 610225, China; huangrc9795@163.com (J.H.); lixiaojie000000@163.com (X.L.); xi.wu@cuit.edu.cn (X.W.)

**Keywords:** precipitation nowcasting, radar-satellite fusion, multi-scale modeling, frequency-domain representation

## Abstract

Accurate precipitation nowcasting plays an important role in disaster prevention and hydrometeorological applications, yet it remains highly challenging due to the complex spatiotemporal variability and multi-scale structural characteristics of precipitation systems. Existing deep learning methods are largely data-driven and often struggle to effectively exploit multi-source observations or learn physically meaningful representations. To address these limitations, this study proposes a Multi-Scale Frequency–Temporal Network (MS-FTNet) for precipitation nowcasting. The framework leverages Fourier transform-based frequency-domain modeling to achieve an interpretable multi-scale decomposition of precipitation dynamics. Specifically, low-frequency components capture large-scale stratiform patterns and their temporal evolution, while high-frequency components represent localized convective structures and abrupt variations. Building on this, a Global Feature Collaboration (GFC) module integrates global frequency-domain representations with multi-scale convolutional features, and an Adaptive Temporal Fusion (ATF) module enhances temporal dependency modeling. Experiments on the SEVIR dataset demonstrate that MS-FTNet consistently outperforms representative baseline models in terms of MSE, CSI, and LPIPS, particularly for heavy precipitation events and longer forecast lead times.

## 1. Introduction

Precipitation nowcasting is a critical component of the weather forecasting system and plays an important role in disaster prevention and mitigation, urban operation management, and public safety, among other applications [1,2,3]. However, its forecast accuracy and robustness still leave substantial room for improvement due to multiple limiting factors [4]. On the one hand, precipitation processes exhibit strong nonlinearity, randomness, and multiscale complexity. Their evolution is often rapid and accompanied by sudden changes and significant uncertainty, which makes it highly challenging to accurately characterize their spatiotemporal dynamics [5]. However, current operational weather forecasting still relies predominantly on physics-based Numerical Weather Prediction (NWP) models [6,7]. In the short term, rapidly updating nowcasting scenarios, NWP methods increasingly reveal limitations in terms of computational cost, timeliness, and their ability to resolve small- and medium-scale precipitation systems [8]. In addition, traditional extrapolation-based nowcasting approaches suffer from evident deficiencies in fully exploiting historical information, extending forecast lead times, capturing fine-scale structural details, and effectively integrating multi-source observational data [9], such as radar and satellite measurements [10].

With the rapid development of artificial intelligence technologies, deep learning methods have demonstrated significant advantages in precipitation nowcasting owing to their powerful feature learning and nonlinear modeling capabilities, leading to a series of breakthrough advances in this field [11,12]. For example, Wang et al. [13] proposed the Memory In Memory (MIM) model, which explicitly models higher-order temporal difference information by introducing multi-level memory structures into recurrent neural networks, thereby effectively alleviating the problems of gradient vanishing and memory degradation. Shi et al. [14] introduced the TrajGRU model, which incorporates a learnable dynamic connection mechanism, enabling the network to adaptively capture common non-rigid motion patterns in precipitation echoes, such as translation, rotation, and deformation, thus enhancing its ability to represent complex spatiotemporal evolution processes. Building on this line of work, Wang et al. [15] further proposed the PredRNNv2 model, which strengthens decoupled modeling and effective transmission of spatiotemporal information by introducing causal temporal constraints and a memory decoupling mechanism, thus improving the stability and consistency of long-sequence predictions to some extent. In addition, the MetNet-3 model proposed by Espeholt et al. [16] adopts an end-to-end deep learning framework to jointly model radar, satellite, and multiple meteorological variables, achieving notable progress in multi-source meteorological data fusion and model generalization performance. The STCINet model developed by Ali et al. [17] enhances causal modeling capability and the interpretability of predictions by introducing causal convolutions and spatial context encoding mechanisms.

Precipitation is inherently the result of coupled multi-scale physical processes, with its spatial organization and temporal evolution profoundly influenced by atmospheric dynamic mechanisms. However, most existing deep learning-based precipitation nowcasting models still rely primarily on purely data-driven paradigms and lack explicit characterization of the multi-scale physical features of precipitation. In addition, some models (such as PredRNNv2 and MIM) mainly depend on single-source data, making it difficult to simultaneously achieve high resolution, broad coverage, and the integration of multi-physical information. Recurrent neural network (RNN)-based models, represented by PredRNNv2 and TrajGRU, adopt an autoregressive approach to generate predictions frame by frame, where small deviations in early predictions are progressively amplified through the recurrent architecture, leading to accumulating errors in long-term forecasts. These limitations cause prediction errors to accumulate rapidly as the forecast lead time increases, making it difficult for existing models to deliver stable and reliable forecasts in operational settings [18,19].

Based on the aforementioned issues, this study posits that integrating multi-source observational data with modeling strategies capable of characterizing the physical evolution of precipitation is a key approach to enhancing the performance of deep learning-based precipitation nowcasting. Precipitation observational data mainly comprise radar and satellite measurements. Radar provides high-precision, high spatiotemporal resolution three-dimensional structures of precipitation but has limited coverage, whereas satellites offer extensive, continuously covered cloud information that compensates for radar’s observational gaps. The fusion of these two data sources facilitates a spatiotemporally continuous characterization of precipitation evolution, which is particularly critical for rapidly evolving hazardous weather events such as short-duration heavy rainfall. Among the various methods for characterizing multi-scale physical features, the Fourier transform, as a classic tool in physics and signal analysis, can reveal global correlation structures and potential periodic evolution patterns in spatiotemporal fields from a frequency-domain perspective, thereby providing a natural and effective framework for modeling precipitation evolution. However, relying solely on frequency-domain modeling is insufficient to fully capture the local details and non-stationary variations of precipitation fields. Therefore, it is necessary to adopt a multi-module collaborative design that integrates Fourier-based frequency-domain modeling with spatial local feature learning and temporal dynamic modeling.

Inspired by the EarthFarseer [20] model, we propose a Multi-Scale Fourier Temporal Network (MS-FTNet) to enhance the modeling capability and prediction accuracy of complex precipitation evolution processes by incorporating Fourier transform-based modeling and integrating multi-source remote sensing observations from satellites and radar. MS-FTNet incorporates Fourier-based frequency-domain modeling at two critical stages: spatial feature extraction and temporal evolution modeling. This design establishes a unified framework that integrates global frequency-domain representations with local spatial feature learning and temporal dynamic modeling, enabling complementary characterization of precipitation evolution across multiple spatial and temporal scales while effectively bridging global dependencies and local structural details.

Specifically, the Fourier-based Computing Unit (FCU) [21] performs spectral decomposition of spatiotemporal features via a two-dimensional Fourier transform, mapping precipitation information into the frequency domain. In the spatial dimension, low-frequency components correspond to the background field and advection processes of large-scale stratiform precipitation, while high-frequency components capture the boundaries and textural details of localized convective cells. In the temporal dimension, low-frequency components characterize the persistent evolution of precipitation systems, whereas high-frequency components depict nonlinear variations such as convective initiation and dissipation. By integrating the global frequency-domain features extracted by the Fourier-based modules with the local features derived from other modules, the model effectively captures both the macroscopic trends and fine-grained local evolution of precipitation, thereby enhancing forecast accuracy.

In the spatial feature extraction stage, this paper adopts a frequency-spatial collaborative modeling strategy (GFC). This module consists of two branches, namely the Global Frequency-Domain Modeling Block (GF-Block) and the CNN Branch, which are coordinated through multi-level fusion. The GF-Block is primarily responsible for modeling global spatial dependencies across regions, while a parallel CNN Branch focuses on capturing local texture structures and fine-grained details. Through multiple rounds of bidirectional interaction and feature fusion, the model effectively integrates contextual information in the global frequency domain while preserving local spatial structural consistency, thereby alleviating the inherent limitations of conventional convolutional networks in modeling long-range spatial dependencies.

In the temporal modeling stage, this paper proposes an Adaptive Temporal Fusion (ATF) module to integrate temporal and frequency-domain information. This module consists of two parallel submodules, namely the Multi-Scale Fourier Module (MSFN) and the Adaptive Fusion Module (AF). At this stage, the MSFN module introduces the FCU to characterize long-term evolutionary trends and potential periodic patterns in time series from a frequency-domain perspective. Meanwhile, the AF branch (which comprises an AvgPool layer and a fully connected layer) performs global statistical modeling along the temporal dimension, serving as an effective complement to the frequency-domain temporal modeling module. This cooperative design allows frequency-domain representations to retain fine-grained temporal dynamics while effectively avoiding excessive smoothing of high-frequency information as the prediction horizon extends.

Extensive experiments on the SEVIR dataset demonstrate that the proposed MS-FTNet consistently outperforms representative baseline models in precipitation nowcasting tasks, suggesting the effectiveness of integrating frequency-domain global modeling with multi-scale spatiotemporal feature learning, particularly for structurally complex and heavy precipitation events.

The main contributions of this work are summarized as follows:

- We propose MS-FTNet, a multi-scale frequency–temporal network for precipitation nowcasting that effectively fuses heterogeneous satellite infrared and radar VIL observations. The framework consists of four core components: a dual encoder for source-specific feature extraction, a Global Feature Collaboration (GFC) module for frequency-enhanced spatial modeling, an Adaptive Temporal Fusion (ATF) module for frequency-domain temporal dependency learning, and a decoder for reconstructing precipitation predictions. The overall architecture enables coordinated modeling of multi-source spatiotemporal information.
- We introduce a unified spatiotemporal feature modeling module that jointly integrates information from the spatial, temporal, and frequency domains. Through a multi-branch collaborative mechanism, the model achieves complementary modeling of local details and global structural information, providing effective feature representations for high-accuracy precipitation nowcasting.
- Comprehensive experimental evaluations on the SEVIR dataset [22] demonstrate that our proposed MS-FTNet consistently outperforms existing mainstream methods across multiple evaluation metrics, validating its superior predictive accuracy, stability, and generalization capability in precipitation nowcasting tasks.

## 2. Data

### 2.1. The Storm EVent ImagRy Dataset (SEVIR)

The SEVIR (Storm EVent ImageRy) dataset is a high-resolution meteorological dataset designed for deep learning research. It contains more than 10,000 weather events, each covering a spatial region of approximately 384 km × 384 km, and provides continuous multi-source, temporally aligned observation sequences spanning 4 h with a temporal resolution of 5 min. The dataset integrates multiple types of remote sensing observations, including satellite and radar data, and has been widely used in meteorological analysis and short-term precipitation nowcasting studies. As shown in Table 1, this study utilizes two infrared satellite channels (IR069 and IR107) and the vertically integrated liquid water (VIL) data from the SEVIR dataset. The SEVIR dataset and its detailed documentation are publicly available through the AWS Open Data Registry, maintained by the National Oceanic and Atmospheric Administration (NOAA), at https://registry.opendata.aws/sevir/ (accessed on 23 December 2025).

### 2.2. Dataset Construction

For the precipitation nowcasting task, multi-source remote sensing observations from the past 1 h are used as model inputs, while precipitation information for the subsequent 1 h is taken as the prediction target. Specifically, the input variables include two infrared satellite channels (IR069 and IR107) and VIL data, and the prediction target is the single-channel VIL at future time steps. The data used in this study span from January to May 2019. The overall construction process of the dataset is as follows.

#### 2.2.1. Data Processing

First, invalid values in the infrared satellite data (IR069 and IR107) are removed, and the brightness temperature is converted to physical quantities. Next, samples with an excessively high proportion of invalid values or obvious quality anomalies are discarded. Finally, considering that different observation methods have different spatial resolutions, all data frames are uniformly resampled to a spatial resolution of 128 × 128 using bilinear interpolation.

#### 2.2.2. Spatiotemporal Sample Construction

**Figure 1:**
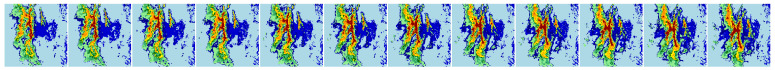
Example of SEVIR data sequences, where each sequence consists of 12 frames.

In the SEVIR dataset, each weather event lasts for 4 h, corresponding to 48 observation frames. A sliding window strategy with a window length of 24 frames and a step size of 12 frames is adopted to construct spatiotemporal sequence samples, where the first 12 frames (1 h) are used as the input sequence, and the subsequent 12 frames are used as the prediction sequence (as shown in Figure 1). As a result, three spatiotemporal samples can be generated from each event, ensuring that the precipitation patterns in each sample are not duplicated. Ultimately, 7440 spatiotemporal samples are generated. The input sequence consists of three channels (IR069, IR107, and VIL), while the output sequence contains single-channel VIL data for the next 12 frames.

#### 2.2.3. Dataset Partitioning

To prevent potential data leakage, among the three samples generated from the same event, the temporally intermediate sample was preferentially assigned to the test set, while the remaining samples were randomly shuffled and allocated to the training and test sets. Ultimately, the ratio of the training, validation, and test sets was set to 7:1:2. This strategy aims to maximize the number of available samples while avoiding information leakage caused by temporal correlations.

## 3. Method

To characterize the multi-scale features of precipitation systems in terms of spatial structure and temporal evolution, and to effectively integrate the global frequency-domain information involved in precipitation processes, this study proposes a multi-source data-driven Multi-Scale Fourier Temporal Network (MS-FTNet).

**Figure 2:**
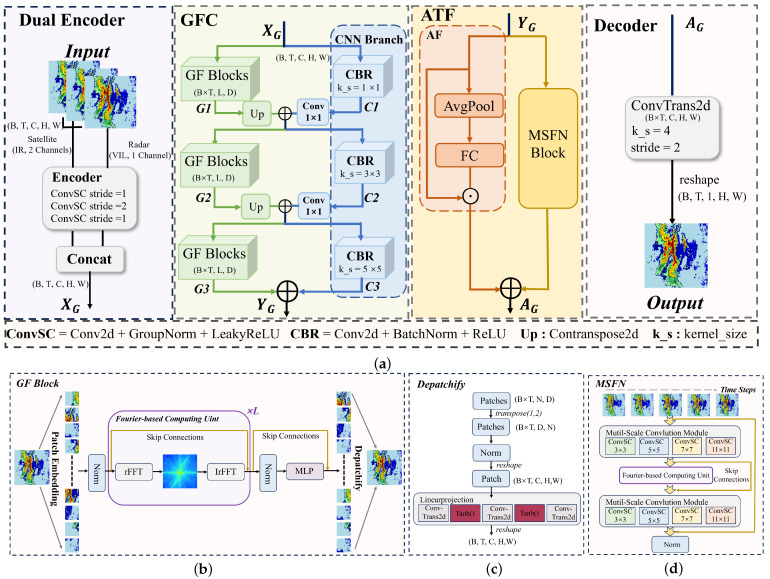
Framework of MS-FTNet. The light green regions denote the Global Feature Collaboration (GFC) module, while the light yellow regions correspond to the Adaptive Temporal Fusion (ATF) module. (**a**) presents the overall architecture of MS-FTNet; (**b**) shows the detailed structure of the GF-Block within the GFC module; (**c**) further illustrates the Depatchify operation inside the GF-Block shown in (**b**); and (**d**) depicts the detailed structure of the MSFN module in the ATF framework.

As shown in Figure 2a, the overall architecture of MS-FTNet consists of four components: dual-encoder multi-source representation learning, a Global Feature Collaboration (GFC) module for frequency-enhanced spatial modeling, an Adaptive Temporal Fusion (ATF) module for frequency-domain temporal dependency learning, and a decoder for reconstructing precipitation predictions. Figure 2b illustrates the structure of the GF-Block within the GFC module shown in (a), Figure 2c presents the detailed architecture of Depatchify in (b), and Figure 2d shows the structure of the MSFN within the ATF module in (a). The detailed implementation of each component will be elaborated in the following sections.

### 3.1. Dual-Encoder for Multi-Source Representation Learning

Satellite infrared observations and radar Vertically Integrated Liquid Water (VIL) measurements exhibit fundamental differences in terms of observation geometry, physical sensitivity, spatial continuity, and noise characteristics. Satellite infrared data primarily represent the thermodynamic and radiative properties of cloud tops with broad spatial coverage, whereas radar VIL focuses on the vertical integration of precipitation intensity, featuring higher spatial variability and localized measurement uncertainty. Directly concatenating these two heterogeneous inputs at the raw data level may lead to feature confusion and hinder the model’s ability to effectively distinguish the physical information inherent to each observation source.

To address this issue, we introduce a dual-encoder architecture that independently models satellite and radar observations before feature fusion. Each encoder consists of three stacked ConvSC blocks, with each ConvSC block comprising a 2D convolutional layer, group normalization, and a LeakyReLU activation function. Although the two encoders share the same network architecture, their parameters are learned independently, enabling each branch to adaptively extract feature representations that align with the physical characteristics of its respective data source.

After source-specific feature extraction, the encoded satellite and radar features are concatenated along the channel dimension to form a unified spatiotemporal representation. This late fusion strategy preserves the distinct physical semantics of each observation type while allowing complementary information to be integrated at a higher semantic level. The resulting feature representation provides a robust and physically meaningful foundation for subsequent global frequency-domain modeling and spatiotemporal evolution learning in the GFC module.

### 3.2. Fourier-Based Computing Unit (FCU)

As shown in Figure 2b, the proposed Fourier-based Computation Unit (FCU) is designed as a global modeling operator for high-dimensional spatiotemporal features, aiming to explicitly capture long-range dependencies through frequency-domain transformation. Conventional convolution operations are inherently limited by local receptive fields, making it difficult to effectively model global interactions between distant spatial locations or time steps. In contrast, the FCU transforms feature representations into the frequency domain, where global interactions can be modeled in a unified and computationally efficient manner, thereby compensating for the limitations of convolution operations in global perception.

Within the MS-FTNet framework, the FCU serves as a core frequency-domain modeling primitive and is embedded into both the spatial modeling module (GFC) and the temporal modeling module (ATF), adopting task-oriented feature reorganization strategies tailored to different modeling objectives. Specifically, in the GFC module, the FCU merges the batch and temporal dimensions (B×T) to perform unified global spatial modeling of the two-dimensional precipitation field independently at each time step. This design enhances the model’s ability to capture large-scale spatial organization and the overall structure of precipitation systems. In the ATF module, the FCU merges the temporal and channel dimensions (T×C), enabling joint modeling of multi-temporal-scale feature representations in the frequency domain. Through this time–frequency coupled modeling approach, the FCU explicitly captures long-range dependencies across different time steps and temporal scales, thereby enabling a more accurate characterization of precipitation evolution dynamics.

The detailed architecture and computational workflow of the FCU are described as follows. Its core objective is to perform global modeling of high-dimensional features in the frequency domain, explicitly capturing long-range dependencies between distant spatial locations or time steps. The overall computational process consists of four stages: feature reorganization, frequency-domain transformation, linear operations in the frequency domain, and inverse transformation for feature reconstruction.

Given an input feature $X\in R^{B\times N\times C}$ (where N=h×w; *B*, *C*, *h*, and *w* represent the batch size, channel number, height, and width, respectively), it is first reshaped into a spatial grid representation $\tilde{X}\in R^{B\times h\times w\times C}$. Subsequently, a two-dimensional fast Fourier transform (FFT) is applied to real-valued feature maps, mapping the feature representations from the spatial domain into the frequency domain.

(1) $$F=F_{2}\left(\tilde{X}\right),\;F\in C^{B\times h\times \left(\frac{w}{2}+1\right)\times C}.$$

Here, $F_{2}\left(\cdot \right)$ denotes a two-dimensional fast Fourier transform (FFT) applied to real-valued feature maps. This transformation projects spatial features from the spatial domain into the frequency domain, where global spatial dependencies can be represented explicitly through spectral components. By operating in the frequency domain, long-range interactions among distant spatial locations can be efficiently modeled, providing a suitable basis for subsequent global feature transformation.

In the frequency domain, an adaptive Fourier operator $G_{\theta }$ is introduced to transform spectral features and enable explicit global interaction modeling. Specifically, $G_{\theta }$ applies learnable linear transformations to the real and imaginary components of the complex-valued spectrum independently, followed by nonlinear activation to enhance representational capacity:

(2) $$G_{\theta }\left(F\right)=\phi \;\left(W_{1}F+b_{1}\right)W_{2}+b_{2},$$

where $W_{1}$ and $W_{2}$ denote learnable spectral weight matrices; $b_{1}$ and $b_{2}$ are bias terms; and ϕ(·) represents the ReLU activation function. This adaptive spectral transformation enables the model to flexibly modulate the contributions of different frequency components, thereby enhancing its ability to capture globally coherent structural patterns in the frequency domain.

After spectral transformation, the frequency-domain features are projected back to the spatial domain through a two-dimensional inverse fast Fourier transform (IrFFT) applied to the complex-valued spectrum. The reconstructed spatial features are then reshaped into a sequential representation consistent with the original input format. To further enhance feature expressiveness, a learnable channel-wise bias is introduced, allowing the model to adaptively recalibrate feature responses across channels. In addition, a residual connection with the input sequence X is employed to preserve original feature information and stabilize optimization. Through this design, the FCU effectively balances global dependency modeling with local feature fidelity.

(3) $$Y=X+\left(Reshape\;\left(F_{2}^{-1}\left(G_{\theta }\left(F\right)\right)\right)+bias\right),\;Y\in R^{B\times N\times C}$$

where $F_{2}^{-1}\left(\cdot \right)$ denotes the inverse Fourier transform and bias represents a learnable channel-wise bias term.

Through the above design, the FCU enables efficient and explicit modeling of global dependencies while preserving the original spatial resolution. Compared with global modeling approaches based on self-attention, the FCU achieves global interaction modeling with lower and more controllable computational complexity, making it particularly suitable for learning high-resolution spatiotemporal features. Benefiting from its flexible feature dimension reorganization mechanism, the FCU serves as a unified frequency-domain modeling primitive. It is employed for global frequency-domain spatial modeling in the GFC module and for frequency-domain temporal evolution modeling in the ATF module. By adaptively reorganizing feature dimensions according to different modeling objectives, the FCU provides globally aware feature representations for subsequent processing stages, thereby enabling the unified characterization of spatial structural consistency and long-range temporal dependencies within a single framework.

### 3.3. Global Feature Collaboration Module (GFC)

To effectively leverage the complementary characteristics of satellite and radar observations in precipitation forecasting, this paper designs a Global Feature Collaboration (GFC) module, which adopts a dual-branch parallel architecture consisting of a global frequency-domain modeling branch (GF-Block) and a convolutional neural network branch (CNN Branch).

The GF-Block aims to model the large-scale organizational structure and long-range spatial dependencies of precipitation systems through frequency-domain feature interaction. By performing block-wise global feature extraction, this branch effectively captures the overall evolution patterns of precipitation systems, such as the continuity of rain belts and the structural consistency at the system scale. However, frequency-domain modeling is relatively insensitive to local intensity variations and sharp spatial gradients, which are crucial for representing convective cores and fine-scale precipitation structures.

To compensate for this limitation, the CNN Branch is introduced to preserve and enhance local spatial details. This branch extracts local precipitation features under different receptive fields through multi-scale convolutional modules, thereby effectively characterizing fine-scale structures such as small-scale convective cells and intensity gradients.

Feature interaction between the two branches is achieved through a multi-stage collaboration mechanism. At each stage, the global features output by the GF-Block are progressively upsampled to match the spatial resolution of the CNN Branch features. After channel-wise adaptation via 1×1 convolutions, the global features are fused with local features through element-wise addition, allowing the global contextual information to effectively guide the refinement of local features. Through repeated feature exchanges across multiple stages, the GFC module ultimately learns a unified spatiotemporal representation that maintains global structural coherence while preserving local precipitation details, making it highly suitable for modeling the multi-scale and highly nonlinear characteristics inherent in precipitation evolution.

#### 3.3.1. Global Frequency-Domain Modeling Block (GF-Block)

As illustrated in Figure 2b, in the GF-Block, the input feature $X_{G}$ is first rearranged and reshaped to ${\hat{X}}_{G}\in R^{(B\times T)\times C\times h\times w}$. Subsequently, a Patch Embedding operation is applied to partition and project local spatial features into a sequence of token representations, thereby providing a structured input for subsequent frequency-domain global modeling:

(4) $$X_{P}=\mathrm{PatchEmbed}\left({\hat{X}}_{G}\right)\in R^{(B\times T)\times N\times D},\;N=h\times w$$

where *D* denotes the embedding dimension, and (h,w) represents the patch grid size.

To preserve spatial structural information, a learnable position embedding is introduced into the token sequence and added to the input features to obtain P. The resulting features are then successively processed by *L* stacked Fourier-based Computing Units (FCUs), enabling global frequency-domain modeling and producing the final frequency-enhanced representation $P_{L}$. After the frequency-domain modeling stage, a normalization layer followed by a multilayer perceptron (MLP) is used to further transform the nonlinear features along the channel dimension. Both modules are equipped with residual connections to improve the capability of presenting features and improve the stability of the training, resulting in the final output feature $\hat{P}$:

(5) $$\begin{array}{cc} P & =X_{p}+E_{pos},P\in R^{(B\times T)\times N\times D}\\ P_{l+1} & =X_{l}+FCU\left(X_{l}\right),l=0,\ldots ,L-1\\ \hat{P} & =Norm\left(MLP\left(P_{L}\right)\right),\hat{P}\in R^{(B\times T)\times N\times D} \end{array}$$

where $E_{pos}$ denotes the learnable positional embedding used to encode spatial location information at the patch level, *L* represents the number of FCU layers stacked, *D* denotes the embedding dimension, and N=h×w indicates the number of patches determined by the spatial patch grid.

As illustrated in Figure 2c, the serialized features $\hat{P}$ are then reshaped back into 2D spatial feature maps:(B×T,N,D)→(B×T,C,h,w). Subsequently, the low-resolution multi-channel features are progressively upsampled through a linear projection module implemented with transposed convolutions, restoring the target spatial resolution and channel dimension. The final output of the GF-Block is denoted as $G_{i}\left(i=1,2,3\right)$.

#### 3.3.2. CNN Branch

To effectively capture the local-scale structural characteristics of precipitation fields, a multi-scale convolutional architecture is adopted for local feature modeling. Specifically, multiple CBR modules (Convolution–Batch Normalization–ReLU) with different kernel sizes (1×1, 3×3, and 5×5) are used to construct hierarchical local feature representations $C_{1},C_{2},C_{3}$. Through progressively expanding spatial receptive fields, these multi-scale features enable the model to comprehensively capture local intensity variations and fine-grained structural details of precipitation echoes at different spatial scales.

### 3.4. Adaptive Temporal Fusion Module (ATF)

The evolution of precipitation systems exhibits significant spatiotemporal heterogeneity, with different historical time steps contributing differently to future precipitation development. Assigning uniform importance to all time steps may obscure the critical transition stages in precipitation evolution, thereby limiting the model’s ability to capture dynamic change processes.

To address this issue, we propose the Adaptive Temporal Fusion (ATF) module, which aims to explicitly model the importance of different time steps while simultaneously capturing long-range temporal dependencies in the frequency domain. As illustrated in Figure 2a, the ATF module consists of two functionally complementary sub-branches: (i) Adaptive Fusion (AF) branch: This branch employs a dynamic weighting mechanism along the temporal dimension to adaptively evaluate the contribution of each historical time step to the current prediction, highlighting critical turning points and transition stages in precipitation evolution. (ii) Multi-scale Fourier-based Temporal Modeling (MSFN) branch: This branch jointly models multi-temporal-scale features in the frequency domain, effectively capturing long-range dependencies across different time steps and characterizing the continuous evolution dynamics of precipitation systems.

The outputs of the two branches are fused via element-wise addition to form a unified temporal representation. This design accounts for both the varying importance of individual time steps and the global modeling of long-range temporal dependencies, enabling the model to achieve a more comprehensive understanding of the temporal evolution patterns in precipitation systems.

#### 3.4.1. Adaptive Fusion Module (AF)

The AF module aims to adaptively emphasize the relative importance of different temporal states. Given an input feature sequence, global average pooling is first applied along the spatial dimensions to obtain a compact temporal descriptor. This descriptor is then transformed through a lightweight fully connected network to generate channel-wise temporal weights.

#### 3.4.2. Multi-Scale Fourier Module with Normalization Module (MSFN)

As illustrated in Figure 2d, in the MSFN, the temporal and channel dimensions are first flattened and fused, resulting in a unified feature representation $X\in R^{B\times (T\times C)\times H\times W}$. This fusion enables joint modeling of temporal dynamics and channel-wise information within a unified spatial framework.

The fused features are then fed into the first Multi-Scale Convolution Module, which is composed of multiple convolutional sub-layers with progressively increasing kernel sizes (*kernel_size* = 3, 5, 7, 11). Without altering the spatial resolution, this module captures precipitation evolution patterns at multiple receptive-field scales, facilitating the extraction of both fine-grained local details and broader structural characteristics.

To preserve multi-level temporal representations, the outputs of the first three convolutional sub-layers are retained as skip connections for subsequent feature recovery. Specifically, the output of the *i*-th convolutional sub-layer in the encoder is connected to the $\left(N_{T}-i\right)$-th sub-layer in the decoder, where $N_{T}=4$ and i=1,2,3 in this study.

The intermediate features are subsequently processed by the Fourier-based Computing Unit (FCU) to perform global frequency-domain modeling, enabling explicit capture of long-range dependencies across temporal and spatial dimensions. After frequency-domain enhancement, a second Multi-Scale Convolution Module is employed to refine local spatial details and recover structural information. Finally, a normalization layer is applied to stabilize feature distributions and facilitate efficient training.

## 4. Experiments

### 4.1. Experimental Settings

#### 4.1.1. Compared Methods

To evaluate the performance of the proposed MS-FTNet framework, we conducted extensive experiments in this section and compared MS-FTNet with several state-of-the-art models, including MMVP [23], SSA-UNet [24], PredRNNv2 [15], SimVP [25], TAU [26], and STCINet [17].

To ensure a fair comparison, all experiments were conducted under the same conditions using the PyTorch 1.8.0 framework and an NVIDIA GeForce RTX 4090 GPU (NVIDIA, Santa Clara, CA, USA). For all compared methods, if the official open-source code provides specific hyperparameter settings, we directly adopt those settings; otherwise, we uniformly adopt the same hyperparameter configuration as the MS-FTNet model proposed in this paper to ensure consistency in experimental conditions.

#### 4.1.2. Evaluation Metrics

The evaluation metrics adopted in this study include the widely used Mean Squared Error (MSE) [27], Peak Signal-to-Noise Ratio (PSNR) [28], Critical Success Index (CSI) [29], and Learned Perceptual Image Patch Similarity (LPIPS) [30]. These metrics provide a comprehensive assessment of model performance from multiple perspectives, including pixel-level accuracy, structural fidelity, event detection capability, and perceptual similarity.

Specifically, MSE measures the average squared difference between predicted and ground truth pixel values and is sensitive to large deviations. It is widely used to evaluate regression accuracy in precipitation nowcasting tasks.

PSNR, derived from MSE, evaluates reconstruction quality from a signal-to-noise perspective. In precipitation nowcasting, PSNR reflects the model’s ability to preserve the spatial distribution and intensity structure of the VIL field. Higher PSNR values indicate that the predicted results are closer to the ground truth in terms of both intensity magnitude and spatial organization, which is essential for accurately representing the spatial patterns and evolution of precipitation systems.

CSI is employed to evaluate the model’s capability in detecting precipitation events under different intensity conditions. In this study, Vertically Integrated Liquid (VIL) is used as a proxy for precipitation intensity due to its strong correlation with convective strength and rainfall intensity. Based on this, multiple VIL thresholds (VIL-20, VIL-30, VIL-40, and VIL-50 kg/m^2^) are adopted to compute CSI, enabling the evaluation of model performance across different precipitation intensity levels, with particular emphasis on moderate-to-heavy precipitation events.

LPIPS measures perceptual similarity by comparing deep feature representations extracted from pretrained neural networks. Unlike pixel-wise metrics such as MSE, LPIPS is more effective in capturing structural and textural differences in precipitation fields, making it more suitable for evaluating the spatial consistency and visual realism of the predicted results.

#### 4.1.3. Implementation Details

The model is optimized using the Adam optimizer with a momentum of 0.9 and a k-decay coefficient of 1.0. The initial learning rate is set to $1\times 10^{-5}$ with a weight decay of 0. The network is trained for a total of 200 epochs with a batch size of 1. The learning rate starts to decay at the 100-th epoch with a decay rate of 0.1, which is dynamically adjusted to a minimum of $1\times 10^{-6}$ via the OneCycle scheduling strategy. To mitigate gradient explosion, L2 norm gradient clipping is employed during training. In addition, the random seed is fixed at 45 to ensure experimental reproducibility.

### 4.2. Experimental Results

As shown in Table 2, this study uses radar-based VIL precipitation data as a benchmark for evaluation. As summarized in the table, the proposed MS-FTNet model demonstrates pronounced advantages in multiple key evaluation metrics. Among the various metrics, MSE and CSI-50 characterize the core performance of precipitation nowcasting models from two complementary perspectives, namely continuous precipitation intensity errors and the capability of detecting heavy precipitation events. Therefore, these two metrics are selected for focused analysis.

Experimental results indicate that, in terms of MSE, MS-FTNet achieves reductions of 34.55%, 30.48%, 22.30%, 69.68%, 34.20%, and 9.87% compared with PredRNNv2 (2.582 $\mathrm{kg}\cdot m^{-2}$), MMVP (2.431 $\mathrm{kg}\cdot m^{-2}$), SimVP (2.175 $\mathrm{kg}\cdot m^{-2}$), STCINet (5.573 $\mathrm{kg}\cdot m^{-2}$), SSA-UNet (2.568 $\mathrm{kg}\cdot m^{-2}$), and TAU (1.875 $\mathrm{kg}\cdot m^{-2}$), respectively. MS-FTNet provides superior accuracy in adjusting the overall precipitation intensity. Regarding the CSI-50 metric, MS-FTNet outperforms PredRNNv2 (0.607), MMVP (0.619), SimVP (0.631), STCINet (0.416), SSA-UNet (0.615) and TAU (0.632) by 9.39%, 7.27%, 5.22%, 59.38%, 8.46%, and 5.06%, respectively. These results indicate that MS-FTNet exhibits a stronger discriminative capability in identifying and detecting heavy precipitation events, leading to a more accurate representation of high-impact precipitation processes.

To further evaluate the model’s performance under different precipitation intensity conditions, the CSI at multiple thresholds (30, 40, 50, and $60\;\mathrm{kg}\cdot m^{-2}$) is computed on the SEVIR dataset, as shown in Table 3. Specifically, the threshold of 30 mainly represents moderate precipitation, thresholds of 40 and 50 correspond to heavy precipitation, while the threshold of 60 characterizes extreme convective events. Overall, the CSI values of all models gradually decrease as the threshold increases. This is because higher thresholds correspond to more complex spatiotemporal evolution processes of intense precipitation, which significantly increases the prediction difficulty. Among all compared methods, MS-FTNet achieves the best performance across all thresholds. Specifically, MS-FTNet reaches 0.664 at CSI-50, outperforming TAU (0.632) by approximately 5.06%. Under the more challenging CSI-60 condition, MS-FTNet (0.426) surpasses TAU (0.397) by about 7.31%. It can be observed that the performance advantage of MS-FTNet becomes more pronounced as precipitation intensity increases, indicating its superior capability in modeling heavy and extreme convective precipitation. In summary, MS-FTNet demonstrates superior performance across different precipitation intensity ranges, suggesting that it can more effectively capture the complex structures and dynamic evolution of precipitation systems.

**Figure 3:**
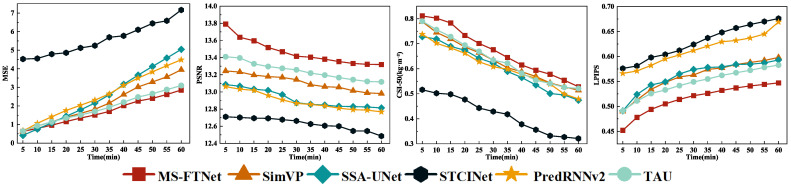
Trend graphs of MSE, PSNR, CSI-50, and LPIPS for each model across multiple time steps, illustrating performance variability and stability.

Figure 3 presents a comparative analysis of MSE, PSNR, CSI-50, and LPIPS for different models with successive prediction lead times. It can be seen that MS-FTNet consistently outperforms all baseline models at every forecast horizon, achieving lower values on error-related metrics (MSE and PSNR) while attaining higher scores on perceptual quality and event-detection metrics (LPIPS and CSI-50). These results indicate that MS-FTNet is more effective in capturing key spatiotemporal structural characteristics of precipitation fields as they evolve over time, demonstrating a clear advantage in identifying and representing high-impact complex weather processes, such as intense convective precipitation systems.

### 4.3. Representative Case Analysis

**Figure 4:**
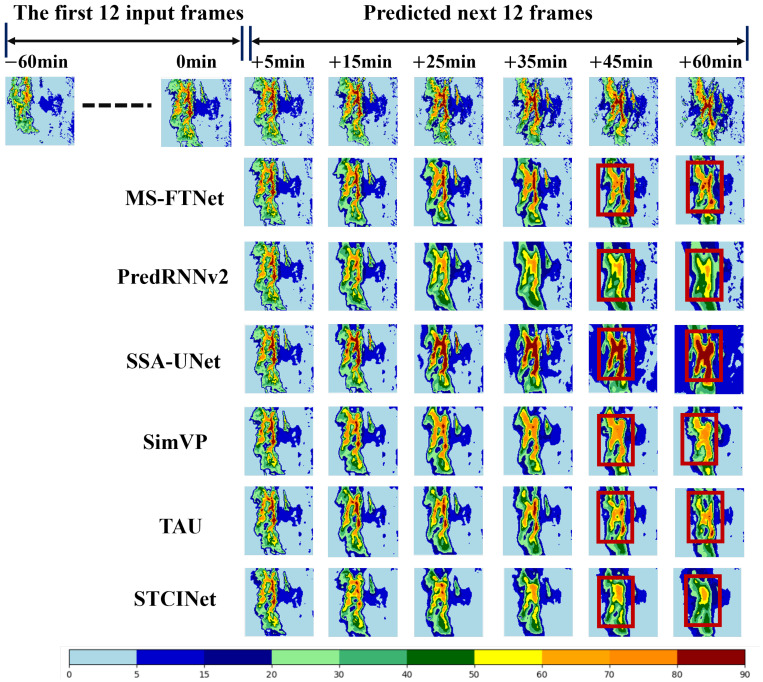
Prediction results of different models for a typical heavy rainfall event.

The dataset used in this study comprises a total of 7440 sample sequences. Among these, samples in which the maximum VIL value in any frame exceeds 60 are defined as heavy rainfall events, yielding a total of 556 such samples. To further evaluate the performance of different models under typical heavy precipitation conditions, this study conducts a case analysis using a heavy rainfall event that occurred between 10:00 and 11:00 on 15 January 2019 in the test set. Figure 4, given the first 12 input frames (−60 min to 0 min), each model predicts the subsequent 12 frames (+5 min to +60 min) in a frame-by-frame manner.

As the forecasting lead time increases, all baseline models exhibit noticeable performance degradation, manifested by blurred precipitation structures, displacement of convective cores, and intensity distortions. These phenomena reflect the significant impact of error accumulation on medium- to long-term nowcasting performance. In contrast, MS-FTNet consistently preserves the spatiotemporal structure and intensity distribution of the precipitation system across all lead times, demonstrating stronger temporal generalization capability and forecasting robustness. Particularly at longer lead times (e.g., +45 min and +60 min), except for MS-FTNet, all other comparison models exhibit obvious intensity distortion (as indicated by the red boxes), with SSA-UNet showing overprediction of large-scale heavy precipitation. This indicates that SSA-UNet is prone to error accumulation over long-term forecasting, leading to substantial deviations between predictions and ground truth. These visual results provide clear evidence that MS-FTNet maintains superior temporal consistency and robustness under extended forecasting horizons, thereby offering improved reliability for continuous precipitation nowcasting tasks.

Table 4 presents the evaluation metrics for this typical case. In terms of pixel-wise fidelity, MS-FTNet achieves the lowest MSE (1.046) and the highest PSNR (12.721), indicating its high reconstruction accuracy. It is worth noting that the MSE performance in this case is better than the average results on the test set shown in Table 4. This is primarily attributed to the presence of a large number of regions with scarce or no precipitation in the test set, where data noise is relatively high, and stability is low. Consequently, even minor prediction deviations can lead to a substantial increase in the metric values. In terms of event detection, MS-FTNet achieves the highest CSI scores across all three thresholds, demonstrating its higher accuracy in capturing heavy rainfall events.

**Figure 5:**
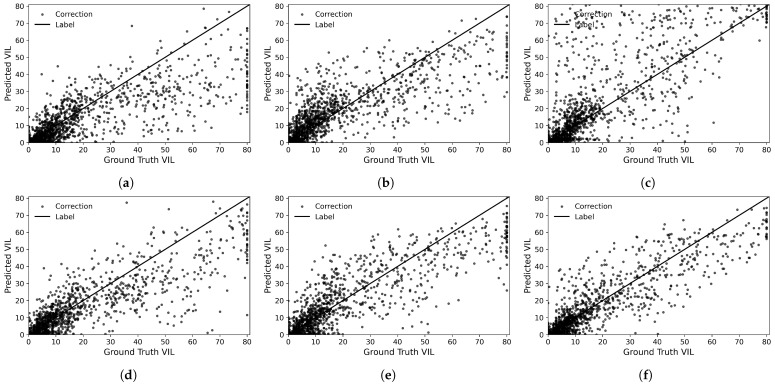
Performance of STCINet, PredRNNv2, SSA-UNet, SimVP, TAU, and our MS-FTNet, under extreme precipitation conditions. (**a**) STCINet. (**b**) PredRNNv2. (**c**) SSA-UNet. (**d**) SimVP. (**e**) TAU. (**f**) Ours.

To further evaluate the predictive performance of different deep learning models under heavy precipitation conditions, a representative rainfall event that occurred at 10:00 on 15 January 2019 was selected for a detailed case-study comparison (see Figure 4). As shown in Figure 5a,b, in regions with relatively low rainfall intensity, STCINet and PredRNNv2 exhibit poorer agreement with the observed precipitation data. Both models show a pronounced tendency to underestimate in areas with high rainfall intensity. Figure 5c indicates that SSA-UNet exhibits an overall overestimation compared to the observed VIL data. This behavior may be attributed to the limited adaptability of its attention mechanism to modeling the complex evolution of precipitation systems, which in turn constrains the model’s ability to learn the spatial distribution characteristics of the intensity of precipitation effectively. As illustrated in Figure 5d–f, when VIL is lower than $20\;\mathrm{kg}\cdot m^{-2}$, the VIL predicted by SimVP, TAU, and MS-FTNet is generally lower than the observed values. However, MS-FTNet produces estimates in high-VIL regions that are closer to the reference line (Label), indicating its superior capability to capture heavy rainfall conditions. In general, MS-FTNet shows the best performance among the models evaluated under strong precipitation scenarios.

### 4.4. Ablation Experiment

#### 4.4.1. The Effect of GFC and ATF Modules

In this section, ablation experiments are conducted to systematically investigate the contributions of the GFC module and the ATF module to the overall performance improvement of the proposed model. Furthermore, the roles of their internal sub-modules—namely, the CNN Branch in the spatial domain and the AF module in the temporal domain—are examined in terms of their ability to assist frequency-domain modeling (i.e., GFC and MSFN) in capturing the spatiotemporal evolution characteristics of precipitation. Specifically, within the GFC spatial feature extraction module, the effects of the GF-Block and the CNN Branch are analyzed, while within the ATF module, the impacts of AF and MSFN are evaluated. To this end, five variants of MS-FTNet are constructed by selectively removing GF-Block, CNN Branch, AF, and MSFN, and their spatiotemporal modeling capabilities are assessed accordingly.

As shown in Table 5, the baseline model (Base), which excludes both the GFC and ATF modules, exhibits the poorest performance across the MSE, PSNR and LPIPS metrics, indicating that relying solely on basic spatiotemporal modeling is insufficient to adequately capture the complex evolution of precipitation fields.

When the GFC-GF module is introduced into the baseline model (Model 1), performance improvements of 37.42% (MSE), 9.11% (PSNR), and 2.17% (LPIPS) are achieved relative to the baseline. This shows that global frequency-domain modeling based on Fourier transforms effectively enhances spatial feature representation.

By further incorporating the CNN Branch into Model 1 to form a complete GFC spatial feature extraction module (Model 2), the performance gains relative to the baseline reach 56.86% (MSE), 12.70% (PSNR), and 7.09% (LPIPS). These results indicate that the CNN Branch compensates, to some extent, for the limitations of block-based frequency-domain modeling in capturing fine-grained local details, thereby improving the completeness of spatial feature representation.

Similarly, introducing only the ATF-MSFN module into the baseline model (Model 3) leads to performance improvements of 31.79% (MSE), 7.59% (PSNR) and 3.18% (LPIPS), validating the effectiveness of multi-scale frequency-domain temporal modeling in characterizing the temporal evolution of precipitation.

Furthermore, when the AF module is added to Model 3 to construct the complete ATF module (Model 4), the performance gains relative to the baseline are further increased to 51.77% (MSE), 10.78% (PSNR) and 4.34% (LPIPS). This suggests that the AF module can adaptively emphasize the relative importance of different time steps, thereby more effectively assisting frequency-domain modeling in capturing the temporal dynamics of precipitation processes.

Model 5 consists solely of GFC-GF and ATF-MSFN, incorporating Fourier-based spatial and temporal feature modeling branches simultaneously. Compared to baseline, this model achieves improvements of 66.33% (MSE), 18.95% (PSNR), and 14.04% (LPIPS), further confirming the effectiveness and representational advantages of frequency-domain modeling in precipitation nowcasting tasks.

Finally, when both the GFC-CNN Branch and ATF-AF are integrated to form the complete MS-FTNet (Model 6), the model achieves the best overall performance, with improvements of 29.72% (MSE), 11.87% (PSNR) and 16.73% (LPIPS) relative to the baseline. These results clearly demonstrate that the GFC and ATF modules, along with their respective sub-modules, exhibit strong complementarity in spatial structure representation and temporal evolution modeling and together constitute key components for enhancing precipitation nowcasting performance.

#### 4.4.2. The Effect of FCU Module

To further investigate the role of the FCU module in precipitation nowcasting, ablation studies are conducted on the FCU components embedded in both the GFC and ATF modules. The corresponding results are presented in Table 4. Specifically, three variants of MS-FTNet are constructed by selectively removing the GFC-FCU and ATF-FCU modules.

As shown in Table 6, Model 1, which excludes both GFC-FCU and ATF-FCU modules, exhibits the worst performance across all three metrics (MSE, PSNR, and LPIPS), indicating that the baseline architecture alone is insufficient to effectively capture the complex spatiotemporal evolution of precipitation. When the GFC-FCU module is introduced into Model 1 (Model 2), the model performance improves significantly, with a notable reduction in MSE, an increase in PSNR, and a decrease in LPIPS. This suggests that incorporating FCU into the GFC module enables the model to capture spatial dependencies from a global frequency-domain perspective, thereby enhancing the representation of overall precipitation structures.

Similarly, when the ATF-FCU module is incorporated into Model 1 (Model 3), all evaluation metrics show improvements compared to Model 1. This demonstrates that the FCU module also plays a critical role in temporal feature modeling, facilitating the capture of dynamic evolution patterns of precipitation systems and improving forecasting accuracy.

Finally, when both GFC-FCU and ATF-FCU modules are integrated to form the complete MS-FTNet, the model achieves the best overall performance. Compared with the baseline model without FCU (Model 1), the MSE is reduced by 52.68%, PSNR is improved by 10.42%, and LPIPS is decreased by 20.52%. These results clearly demonstrate that the FCU module effectively enhances the model’s representation capability in both spatial and temporal dimensions, thereby significantly improving precipitation nowcasting performance.

#### 4.4.3. Ablation Study on Multi-Source Data

To further investigate the contribution of different data sources to precipitation nowcasting performance, we conduct grouped modeling experiments using multi-source fused data, as summarized in Table 7. Specifically, we progressively incorporate infrared satellite observations (IR069 and IR107) together with radar-based VIL data to evaluate their individual and joint impacts.

Group 1 employs only VIL data as input, serving as the baseline multi-source configuration. When IR107 is additionally introduced (Group 2) reduces the MSE by approximately 7.84% and increases CSI-50 by 1.42%, indicating that mid-level infrared observations provide supplementary information to radar-based precipitation intensity estimates.

In comparison, replacing IR107 with IR069 (Group 3) leads to a more substantial improvement, with the MSE reduced by 11.31% and CSI-50 increased by 3.33% relative to the VIL-only baseline. This larger gain suggests that IR069 exhibits a stronger coupling with precipitation-related cloud structures and moisture distributions.

Finally, when all three data sources (IR069, IR107, and VIL) are jointly integrated in MS-FTNet, the model achieves the best overall performance. Compared with Group 1, MS-FTNet achieves a 14.98% reduction in MSE and a 5.40% increase in CSI-50, while also improving perceptual similarity as reflected by a 3.33% decrease in LPIPS.

Overall, the ratio-based analysis clearly demonstrates that (i) infrared information consistently enhances precipitation nowcasting when combined with radar VIL data, (ii) IR069 contributes more effectively than IR107, and (iii) multi-source fusion yields synergistic performance gains that exceed those obtained by any individual or partially fused configuration.

## 5. Discussion

From the overall experimental results, MS-FTNet significantly outperforms current mainstream spatiotemporal prediction models across multiple quantitative metrics on the SEVIR dataset. It exhibits stable and consistent advantages, particularly in two key indicators: MSE and CSI-50. This demonstrates that the proposed multi-scale frequency–temporal joint modeling framework possesses a stronger comprehensive modeling capability to simultaneously characterize precipitation intensity errors and detect heavy precipitation events. Compared with existing methods that rely solely on temporal recurrent structures or local convolutional modeling, MS-FTNet effectively alleviates error accumulation and propagation with increasing forecast lead time by introducing global frequency-domain modeling mechanisms, which is especially evident in medium- and long-range forecasting results.

From the perspective of spatial modeling, the GF-Block based on the Fourier transform within the GFC module plays a critical role in capturing large-scale spatial dependencies, while the multi-scale CNN Branch effectively compensates for the limitations of frequency-domain modeling in representing local fine-grained details. Ablation experiments indicate that introducing either the GF-Block or the CNN Branch alone leads to performance improvements, whereas their joint integration yields the most significant gains. This suggests a strong complementarity between global frequency-domain information and local convolutional features in modeling precipitation spatial structures. This finding aligns with previous studies emphasizing the importance of multi-scale features for precipitation morphology representation, while MS-FTNet further provides a novel frequency-domain perspective for enhancing global spatial consistency.

In terms of temporal modeling, the ATF module fuses the temporal and channel dimensions and performs unified modeling in the frequency domain, enabling the model to explicitly capture global temporal dependencies across different time scales. Experimental results show that the introduction of MSFN alone can significantly improve prediction performance, and further gains are achieved when combined with the AF module. This indicates that the adaptive temporal weighting mechanism effectively emphasizes the varying contributions of different time steps to the prediction. The strategy of “global frequency-domain modeling with adaptive temporal emphasis” facilitates a more stable characterization of precipitation persistence and evolution trends, demonstrating enhanced robustness, particularly in high-impact weather events such as strong convective precipitation.

Moreover, multi-source data ablation experiments further validate the effectiveness of MS-FTNet in information fusion. The results show that infrared satellite observations provide valuable complementary information to radar VIL data, with IR069 exhibiting stronger correlation with precipitation processes than IR107. This phenomenon is physically reasonable, as IR069 is more sensitive to mid- and upper-level cloud structures and water vapor distributions, which are closely related to the development of heavy precipitation. When multi-source data are jointly incorporated, MS-FTNet is able to fully exploit the complementary information among different observations, achieving superior performance compared to single-source inputs or partial fusion configurations.

Despite the significant performance improvements achieved by MS-FTNet in various experimental settings, several issues remain worthy of further investigation. For example, the adaptability of frequency-domain modeling across different spatial resolutions and observation scales, as well as the generalization capability under conditions where extreme precipitation samples are relatively scarce, requires more systematic evaluation. In terms of comparative experiments, MS-FTNet has been evaluated against deep learning forecasting models in the meteorological field, such as SSA-UNet, PredRNNv2, and SimVP. However, due to fundamental differences between traditional numerical weather prediction (NWP) models and deep learning models in terms of input data, physical parameterization schemes, and forecasting mechanisms, it is challenging to conduct fair and rigorous comparative experiments under identical input conditions, spatiotemporal scales, and datasets. Future work will attempt to introduce such traditional models for supplementary comparisons on suitable datasets where conditions permit. Additionally, the current model primarily relies on a data-driven learning approach. How to further incorporate physical constraints into the frequency-domain spatiotemporal modeling framework to enhance interpretability and physical consistency represents an important direction for future research.

Overall, the experimental results and ablation analyses presented in this study demonstrate that multi-scale frequency–temporal joint modeling provides an effective and physically meaningful approach for precipitation nowcasting. This work also lays a solid foundation for extending frequency-domain methods to more complex meteorological spatiotemporal prediction tasks in the future.

## 6. Conclusions

In this study, we propose MS-FTNet, a multi-scale frequency–temporal network for precipitation nowcasting that integrates heterogeneous satellite infrared and radar observations within a unified modeling framework. By combining multi-scale convolutional feature extraction with frequency-domain global dependency modeling, the proposed approach enables coordinated representation of large-scale structural organization and localized precipitation variability. This design improves the capability of the model to capture complex precipitation morphology and long-range spatiotemporal dependencies. Experimental evaluations on the SEVIR dataset indicate that MS-FTNet achieves consistent performance improvements over representative baseline models. In particular, the proposed framework demonstrates an improved ability to preserve the structural coherence of precipitation systems and maintain prediction stability at longer lead times, highlighting the effectiveness of incorporating frequency-domain modeling into precipitation nowcasting tasks.

Despite these promising results, several directions remain open for future research. First, incorporating additional meteorological variables and heterogeneous data sources—such as numerical weather prediction outputs, reanalysis data, terrain elevation, wind fields, and atmospheric parameters—may further enhance the physical consistency, robustness, and generalization capability of the proposed framework, especially for extreme precipitation events. Second, exploring adaptive or learnable frequency selection and decomposition strategies could enable more targeted modeling of scale-dependent precipitation dynamics and further improve the representation of localized abrupt changes. Third, extending MS-FTNet to probabilistic or uncertainty-aware nowcasting frameworks may provide more informative and reliable forecasts for operational applications. Finally, improving computational efficiency and scalability will be essential for deploying frequency-domain spatiotemporal models in real-time precipitation nowcasting systems.

## Figures and Tables

**Table 1:** Summary of multi-source data used in this study.

Image Type	Description	Spatial Resolution	Patch Size	Event Count
ir069	Infrared satellite imagery (mid-level water vapor)	2 km	192 × 192	13,552
ir107	Infrared satellite imagery (clean longwave window)	2 km	192 × 192	13,541
vil	NEXRAD radar mosaic of VIL	1 km	384 × 384	20,393

**Table 2:** Evaluation of MSE, PSNR, CSI-50, and LPIPS on the SEVIR Dataset.

Method	MSE ↓	PSNR ↑	CSI-50 ↑	LPIPS ↓
PredRNNv2	2.582	12.843	0.607	0.613
MMVP	2.431	13.032	0.619	0.608
SimVP	2.175	13.156	0.631	0.547
STCINet	5.573	12.614	0.416	0.652
SSA-UNet	2.568	12.925	0.615	0.608
TAU	1.875	13.245	0.632	0.536
**MS-FTNet**	**1.690**	**13.453**	**0.664**	**0.523**

↓ indicates that lower values are better, and ↑ indicates that higher values are better. The best results are highlighted in bold.

**Table 3:** Evaluation of CSI-30, CSI-40, CSI-50, and CSI-60 on the SEVIR Dataset.

Method	CSI-30 ↑	CSI-40 ↑	CSI-50 ↑	CSI-60 ↑
PredRNNv2	0.692	0.625	0.607	0.316
MMVP	0.708	0.653	0.619	0.357
SimVP	0.716	0.670	0.631	0.383
STCINet	0.561	0.533	0.416	0.289
SSA-UNet	0.702	0.647	0.615	0.337
TAU	0.718	0.674	0.632	0.397
**MS-FTNet**	**0.739**	**0.698**	**0.664**	**0.426**

↑ indicates that higher values are better. The best results are highlighted in bold.

**Table 4:** Evaluation of MSE, PSNR, LPIPS, CSI-40, CSI-50 and CSI-60 on a typical heavy rainfall event.

Method	MSE ↓	PSNR ↑	LPIPS ↓	CSI-40 ↑	CSI-50 ↑	CSI-60 ↑
PredRNNv2	1.982	12.143	0.481	0.462	0.385	0.274
SimVP	1.472	12.351	0.493	0.478	0.417	0.295
STCINet	2.315	12.017	0.464	0.456	0.364	0.261
SSA-UNet	2.868	11.714	0.452	0.438	0.357	0.263
TAU	1.344	12.476	0.506	0.495	0.426	0.317
**MS-FTNet**	**1.046**	**12.721**	**0.524**	**0.516**	**0.453**	**0.348**

↓ indicates that lower values are better, and ↑ indicates that higher values are better. The best results are highlighted in bold.

**Table 5:** Ablation Study on the GFC and ATF Modules for Spatial–Temporal Modeling (on SEVIR Dataset).

Model	Modules				Metrics		
	GFC-GF-Block	GFC-CNN Branch	ATF-AF	ATF-MSFN	MSE ↓	PSNR ↑	LPIPS ↓
Base	–	–	–	–	7.553	10.938	0.691
Model 1	✓	–	–	–	4.727	11.934	0.676
Model 2	✓	✓	–	–	3.258	12.326	0.642
Model 3	–	–	–	✓	5.152	11.768	0.713
Model 4	–	–	✓	✓	3.643	12.117	0.661
Model 5	✓	–	–	✓	2.543	13.011	0.594
**MS-FTNet**	✓	✓	✓	✓	**1.690**	**13.453**	**0.523**

The symbol “✓” denotes that the corresponding module is enabled, whereas “–” indicates that the module is disabled. The arrows ↓ and ↑ indicate that lower and higher values correspond to better performance, respectively. The best results are highlighted in bold.

**Table 6:** Ablation Study on the GFC-FCU and ATF-FCU Modules for Spatial–Temporal Modeling (on SEVIR Dataset).

Model	Modules		Metrics		
	GFC-FCU	ATF-FCU	MSE ↓	PSNR ↑	LPIPS ↓
Model 1	–	–	3.572	12.183	0.658
Model 2	✓	–	2.351	13.165	0.574
Model 3	–	✓	2.645	12.942	0.607
**MS-FTNet**	✓	✓	**1.690**	**13.453**	**0.523**

The symbol “✓” denotes that the corresponding data source is utilized, while “–” indicates that it is not included. ↓ and ↑ indicate that lower and higher values correspond to better performance, respectively. The best values are highlighted in bold.

**Table 7:** Comparative Experimental Results of Grouped Modeling with Multi-Source Fused Data.

Group	Input			Metrics			
	IR069	IR107	VIL	MSE ↓	PSNR ↑	CSI-50 ↑	LPIPS ↓
Group 1	–	–	✓	1.988	13.218	0.630	0.541
Group 2	–	✓	✓	1.832	13.277	0.639	0.531
Group 3	✓	–	✓	1.763	13.429	0.651	0.529
**MS-FTNet**	✓	✓	✓	**1.690**	**13.453**	**0.664**	**0.523**

The symbol “✓” denotes that the corresponding data source is utilized, while “–” indicates that it is not included. ↓ and ↑ indicate that lower and higher values correspond to better performance, respectively. The best values are highlighted in bold.

## Data Availability

The dataset is available at https://registry.opendata.aws/sevir/ (accessed on 23 December 2025).

## Abbreviations

The following abbreviations are used in this manuscript:

MS-FTNet	Multi-Scale Fourier Temporal Network
FCU	Fourier-based Computing Unit
GFC	Global Feature Collaboration
GF-Block	Global Frequency-Domain Modeling Block
ATF	Adaptive Temporal Fusion Module
AF	Adaptive Fusion module
MSFN	Multi-Scale Fourier Module with Normalization Module
